# SASBLS: An Advanced Model for Sleep Apnea Detection Based on Single-Channel SpO2

**DOI:** 10.3390/s25051523

**Published:** 2025-02-28

**Authors:** Yichong She, Di Zhang, Jinbo Sun, Xuejuan Yang, Xiao Zeng, Wei Qin

**Affiliations:** 1 Engineering Research Center of Molecular and Neuro Imaging of the Ministry of Education, School of Life Science and Technology, Xidian University, Xi’an 710071, China; ycshe@stu.xidian.edu.cn (Y.S.); dizhang@stu.xidian.edu.cn (D.Z.); sunjb@xidian.edu.cn (J.S.); xjyang@xidian.edu.cn (X.Y.); wqin@xidian.edu.cn (W.Q.); 2 Guangzhou Institute of Technology, Xidian University, Xi’an 510530, China

**Keywords:** sleep apnea syndrome (SAS), apnea–hypopnea index (AHI), SpO2, broad learning system (BLS)

## Abstract

(1) Background: Sleep Apnea Syndrome (SAS) poses a serious threat to human health. Existing SpO2-based automatic SAS detection models have a relatively low accuracy in detecting positive samples because they overlook the global information from the Apnea–Hypopnea Index (AHI). (2) Methods: To address this problem, we proposed a multi-task model for SAS detection and AHI prediction based on single-channel SpO2. Benefiting from the characteristics of the Broad Learning System (BLS), this model optimizes itself by leveraging the differences between all-night SpO2 information and sample SpO2 information, enabling the two tasks to promote each other. (3) Results: The model was verified using 7906 all-night SpO2 data from the publicly available Sleep Heart Health Study (SHHS) dataset, and the SAS detection performance has reached the state-of-the-art level. In addition, the performance of samples with different lengths in the two tasks was also explored. (4) Conclusions: The model we proposed can balance and effectively perform both SAS detection and AHI prediction simultaneously.

## 1. Introduction

SAS is a common sleep-breathing disorder [1,2] that is widely distributed among various populations. Some demographic characteristics, such as age [3,4], gender [5,6], etc., can generally distinguish the incidence trends of SAS. SAS is characterized by repeated partial or complete blockages of the upper airway during sleep, which leads to oxygen desaturation, sleep disruptions, and a breakdown of normal sleep patterns [7]. SAS patients often experience daytime drowsiness, fatigue, impaired cognitive function, and an increased risk of depression and anxiety [8,9,10]. Severe SAS can lead to a continuous decrease in blood oxygen levels, resulting in insufficient oxygen supply to organs [11]. It is considered to be associated with a variety of diseases [12,13,14], posing a serious threat to an individual’s health. Therefore, it is of great necessity to conduct timely SAS detection.

In the AASM manual [15], the diagnosis of SAS severity is based on the AHI of the subjects. Using the thresholds of 5, 15, and 30, subjects are classified into those without SAS, mild SAS, moderate SAS, and severe SAS. The traditional manual method starts from the judgment of SAS events and calculates the AHI value after statistically analyzing the time information of SAS events, which is a time-consuming and labor-intensive task [16]. Therefore, a large number of researchers have developed automatic AHI estimation models that directly obtain AHI estimates from physiological signals [17] according to the need, skipping the step of SAS event detection. However, in clinical applications, the statistical information of SAS events is still required to further evaluate the subjects’ conditions. Thus, the detection of SAS events in subjects is indispensable [18].

Some researchers, starting from the AASM standards and research accuracy, use multi-channel signals for SAS detection. Manish Sharma et al. proposed a wavelet-based multi-channel signal SAS event detection method [19] by segmenting the signals of respiratory airflow, blood oxygen, thoraco-abdominal belts, and electrocardiogram into 30 s long samples, extracting wavelet features, and using a RUSBoosted tree for event recognition. The model was tested on subjects over 60 years old in the SHHS dataset. The results of SHHS1 showed an accuracy of 83.97%, an MF1 of 61.05%, and a Kappa coefficient of 0.26. The results of SHHS2 showed an ACC of 89.5%, an MF1 of 61.8%, and a Kappa coefficient of 0.26. In Reference [20], the authors extracted features such as sample entropy from the snoring signals, ECG signals, and thoraco-abdominal belt signals of 86 subjects over two nights, and used a support vector machine for OSA event detection. Dandan Peng et al. proposed a bimodal feature fusion CNN model based on nasal pressure airflow and SpO2 signals. The model consists of a dual-branch CNN module and a feature fusion module. The model uses a 10 s time window to segment the signals as input. Tested on 450 samples, the results showed an ACC of 95.91% and an MF1 of 91.38% [21]. Jingliu Luo et al. used the time–frequency diagram of Photoplethysmography (PPG) and SpO2 signals as the input for the system [22], and a graph attention neural network was employed to conduct the OSA detection. When tested on 817 individuals from the Multi-Ethnic Study of Atherosclerosis (MESA) dataset, the accuracy reached 87.03%.

However, considering that the PSG-based acquisition has high requirements for the experimental site and acquisition personnel, and is costly, the application of PSG is also limited [23]. In recent years, Home Sleep Apnea Tests (HSATs) have become popular. Due to their low cost, they have become an effective alternative to PSG [24].

Among them, the SpO2 signal has attracted the attention of many scholars for SAS detection because the sensor is easy to wear, and the change in blood oxygen can directly reflect the impact of SAS events on the human body. In Reference [25], the author proposed an SAS detection model based on a single-channel SpO2 signal and one-dimensional CNN, achieving an ACC of 85.14% on a dataset of 25 subjects. In [26], an electrocardiogram (ECG) and SpO2-based Convolutional Neural Network (CNN) were constructed for OSA detection with 10 s samples. The model was tested on subjects using the Apnea-ECG dataset and the St. Vincent’s University Hospital datasets. Among them, the accuracy of the test results based on SpO2 was 96%. Huijun Yue et al. used a multi-resolution residual network for OAS detection based on a single-channel respiratory airflow signal, with an ACC of 91.2% [27]. Da Woon Jung et al. used a single-channel SpO2 signal, segmented it into 1 min samples, extracted its envelope and event features, and finally used a regression model to regress the AHI and estimate SAS events using the regression results. The accuracy on a test set of 90 subjects was 91.1% [28].

Although existing SAS recognition research has achieved many results, there are still many problems. First, the sample size used in single-channel-based methods is relatively small, and the robustness of the model is difficult to verify. Second, multi-channel-based models have greatly improved in accuracy, but the sample balance is poor. In addition, SAS research based on machine learning methods needs to divide the overnight signals into different samples according to time windows. The sample compositions in existing research are diverse, including 10 s, 30 s, and even 2 min samples, making it lack a unified standard for evaluating model performance.

To solve the above problems, this paper proposes a new SAS event detection model based on a single-channel SpO2 signal. The model innovates in the following aspects:

1. The proposed model realizes two tasks: single-channel SpO2-based SAS event detection and AHI prediction. By fusing the overnight SpO2 features with the sample features, it achieves a mutual promotion between the two tasks.
2. The model uses a single-channel SpO2 signal as input, and its performance exceeds that of existing multi-channel-signal-based models, reaching the state-of-the-art level.
3. The model can effectively identify SAS-positive samples, and its recognition accuracy for SAS-positive samples is much higher than that of other models, matching the application scenarios that require accurate SAS event results.
4. Based on the model, the impact of different sample lengths on the two tasks is explored. To some extent, it solves the problem of non-uniform SAS detection standards.

## 2. Materials and Methods

**Figure 1:**
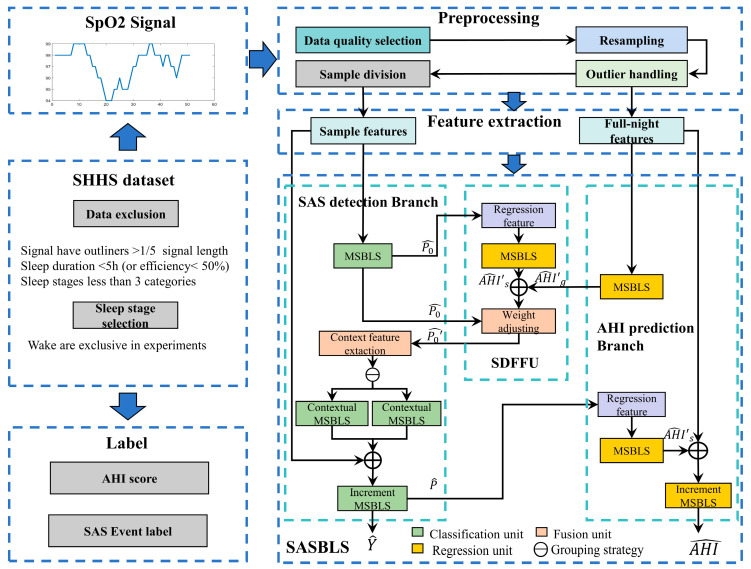
The flowchart of SAS detection and structure of the SASBLS.

The flowchart of SAS event detection is shown in Figure 1. The process consists of four steps: sample screening, pre-processing, feature extraction, and classification. Through these four steps, the system generates outputs, which include the SAS classification result $\hat{Y}$ and the AHI prediction result $\hat{AHI}$. The following will explain these four steps.

### 2.1. Datasets

In this paper, the SpO2 signals from two publicly available datasets, SHHS1 and SHHS2 [29], were used to validate the performance of the model. The demographic information of the dataset used for the experiment is shown in Table 1. The quality of the data has a significant impact on the classification result, and it mainly involves two aspects: 1. Signal quality: When collecting the SpO2 signal, artifacts might appear because of things like changes in the subject’s body position. These signals with artifacts are not suitable for SAS detection. 2. The subject’s sleep quality: This can be considered from the aspects of the subject’s sleep stages and duration. If the subject has too few sleep stages or too short sleep duration, it indicates that the subject has a poor sleep quality and it cannot effectively reflect the subject’s true sleep breathing status. Therefore, the data exclusion criteria are as follows: Firstly, regarding signal quality, if the number of outliers surpasses 1/5 of the signal length, the data of the corresponding subject will be excluded. Secondly, considering the sleep structure, if the polysomnography of a subject exhibits fewer than three sleep stages, the subject is assumed to have sleep disorders and their data will be discarded. Finally, with respect to sleep efficiency, if a subject’s sleep efficiency is lower than 50% or their total sleep time is less than five hours, their data will not be included in the subsequent analysis [17]. Additionally, the *ahi_a0h3a* variable in the dataset is used as the AHI label. The judgment criteria for this variable are as follows: (1) Apnea is defined as a more than 90% decrease in respiratory airflow compared to the baseline, and the duration exceeds 10 s; (2) hypopnea is defined as a 30% decrease in airflow compared to the baseline for more than 10 s, accompanied by a blood oxygen desaturation equal to or more than 3% or an arousal caused by a respiratory event, which is in line with the respiratory event judgment criteria in AASM2.6. The SAS event labels are also selected according to this standard.

### 2.2. Pre-Processing

The pre-processing method of the SpO2 signal is divided into four steps. Firstly, signal clipping is carried out. The SpO2 signals corresponding to the WAKE stage at the beginning and end of the PSG record are removed, and only the SpO2 signals corresponding to the sleep time are retained. Second, down-sampling is performed, and the signal is resampled to 1 Hz. Next, outlier deletion is carried out using subject-wise and sample-wise strategies. For the subject-wise approach, if the outlier duration is under 3 s and outliers account for ≤10% of the sample signal length, they are filtered using a delta filter [17]. Then, sample segmentation is conducted. As shown in Table 1, since the average SAS event length is 20–30 s, the signal and SAS event labels are segmented with a 30 s time window to form samples and labels. For the sample-wise approach, if there is no SAS event in the outlier-corresponding label or the outlier length and proportion do not meet the subject-wise requirements, the sample and its label are deleted. Finally, when processing the PSG labels, only when the length of the event in the SAS time axis corresponding to a sample exceeds 5 s is an SAS event considered to be present in the sample.

### 2.3. Feature Extraction

In this paper, we extract global features from the overnight SpO2 signal and sample features from 30 s samples, respectively. For the global features, we extract 39 features of 5 categories as shown in Table 2, following Reference [17]. In which the time domain features, frequency domain features, and non-linear features are directly extracted from the SpO2 signal. These features effectively mirror the comprehensive information on the SpO2 levels throughout the entire night. The morphological features consider the SpO2 features throughout the whole night as a continuously changing curve and extract the variations in this curve during the whole night. The desaturation events reflect the changes in SpO2 that occur along with the SAS events. The desaturation features conduct statistics on the desaturation events throughout the night, reflecting the overall impact that the SAS events have on SpO2. In addition, demographic factors such as age, BMI, and smoking status are used as supplements. For the sample features, since the sample length is 30 s, and the effective frequency domain features verified by Reference [17] are the power spectral density in the range of 0.03–0.17 Hz, the length of the sample signal does not meet the minimum signal length corresponding to this frequency band. Therefore, it is impossible to extract the frequency domain features of the sample. The same is true for the extraction of morphology and global oxygen desaturation features. Thus, we extract its time domain and non-linear features, specifically including the minimum value, mean value, standard deviation, range value (the maximum value minus the minimum value within the window), the difference between the minimum value and the average value of the overnight SpO2, the percentages of the signal less than 95%, 90%, and 80% in the window length, the sample entropy, and the permutation entropy of the sample signal. In addition, to measure the change in the signal, based on the first-order derivative of the signal, we extract its maximum value, mean value, and the proportion of the part with a change greater than 3% in the signal length. Moreover, taking a 10 s duration with a value 3% lower than the average value of the overnight SpO2 signal as one blood oxygen desaturation event (not following the AASM definition), we calculate the proportion of the desaturation events contained in each sample to the sample length, replacing the desaturation features of the overnight signal.

### 2.4. Model Structure

Based on the inherent characteristics of SAS events in SpO2, we propose a BLS-based SAS event detection model named the SASBLS, as shown in Figure 1. The model adopts a dual-branch structure, consisting of an SAS detection branch based on sample features and an AHI prediction branch based on global features. The SAS branch classifies SAS events by analyzing the features of different samples and the context features among them. The AHI prediction branch predicts AHI based on the features of the overnight SpO2 signal. Furthermore, according to the differences between sample features and global features, the model employs an incremental BLS-based Global Segment Feature Fusing Unit (SDFFU) to optimize the tasks of the two branches, enabling the two tasks to enhance each other and further optimize the model. The specific principle of the model is as follows:

#### 2.4.1. SAS Detection Branch

The SAS detection branch makes a preliminary prediction of SAS events based on sample features and the context features among them. This branch uses an MSBLS [28] as the baseline model to classify sample features for SAS events, obtaining the preliminary SAS prediction probability ${\hat{P}}_{0}$. Then, ${\hat{P}}_{0}$ is transformed through the SDFFU. Considering that SAS events may be divided among different samples, by referring to the method of using context information in Ref. [30], context feature extraction was carried out based on the transformed SAS prediction probability matrix from the SDFFU, and SAS classification based on context features was also performed.

Firstly, the contextual expansion was performed based on the transformed ${\hat{P}}_{0}$. Centering around the current sample, a time domain expansion with a length of 2 is carried out, respectively (150 s in total for the front and back), resulting in the SAS context expansion matrix $P_{con}$, as shown in Equation (1):

(1) $$P_{con}=\left[\begin{array}{ccccc} {\hat{p}}_{-2} & {\hat{p}}_{-1} & {\hat{p}}_{1} & {\hat{p}}_{2} & {\hat{p}}_{3}\\ {\hat{p}}_{-1} & {\hat{p}}_{1} & {\hat{p}}_{2} & {\hat{p}}_{3} & {\hat{p}}_{4}\\ \vdots & \vdots & \vdots & \vdots & \vdots \\ {\hat{p}}_{i-2} & {\hat{p}}_{i-1} & {\hat{p}}_{i} & {\hat{p}}_{i+1} & {\hat{p}}_{i+2}\\ \vdots & \vdots & \vdots & \vdots & \vdots \\ {\hat{p}}_{N-3} & {\hat{p}}_{N-2} & {\hat{p}}_{N-1} & {\hat{p}}_{N} & {\hat{p}}_{N+1}\\ {\hat{p}}_{N-2} & {\hat{p}}_{N-1} & {\hat{p}}_{N} & {\hat{p}}_{N+1} & {\hat{p}}_{N+2} \end{array}\right]$$

Based on $P_{con}$, the context statistical features $P_{cons}$ were extracted, including the number of SAS events in the context corresponding to each sample, the total length, the average length, and the maximum length of the events. Since the locations of SAS events may span multiple samples, in order to examine the concentration degree of SAS events, a continuous length sequence $L_{len}$ was constructed according to the SAS context sequence, as shown in Equation (2):

(2) $$L_{len}=\{\begin{array}{c} 1,\\ 0, \end{array}\begin{array}{c} len\left(i\right)\geq 3\\ len\left(i\right)<3 \end{array}$$

The input of the contextual MSBLS $X_{con}$ was obtained by concatenating the contextual features as $X_{con}=\left[P_{con},P_{cons},L_{len}\right]$. Before the context-based SAS detection, the confidence sequence $C_{SAS}$ needs to be calculated, which was computed by the predicted probabilities of having SAS events as follows (3):

(3) $$C_{SAS}=softmax\left({\hat{P}}_{0}\right)$$

Based on $C_{SAS}$, the samples in the training set and the test set were, respectively, divided into two corresponding groups with a threshold of 0.5. Then, the two groups of data were used for training and testing, respectively. After the results of the two groups were concatenated in order, the contextual features-based SAS detection results were obtained. The results were used as the incremental features and concatenated with the sample feature X1 as the input of the incremental BLS. The outcome of the incremental BLS is the SAS detection results of the model.

#### 2.4.2. SDFFU

Under ideal conditions, the AHI prediction result based on the SAS detection should be the same as that based on global features. However, due to errors in the SAS event detection, there must be a difference between the two AHI prediction values. Conversely, by measuring this difference, the global information can be used to judge the quality of the SAS detection result, thereby optimizing the SAS detection process. The model uses the SDFFU to fuse global features and sample features to optimize this process.

The SDFFU consists of an MSBLS regression model and an SAS prediction probability correction module, where the MSBLS regression model is used for AHI prediction based on the SAS detection result. The input features of the MSBLS regression unit are extracted based on the probability matrix ${\hat{P}}_{0}$ and the classification result ${\hat{Y}}_{0}$ of the first MSBLS unit in the SAS detection branch. These features include the proportion of SAS events in the sleep time of each subject, the average length of events, the standard deviation of lengths, and the proportion of samples with ${\hat{P}}_{0}$ greater than 0.5 in the total length, the proportion of samples with ${\hat{P}}_{0}$ less than 0.3 in the total length, the mean of ${\hat{P}}_{0}$ corresponding to SAS events (i.e., the average probability), and the mean of ${\hat{P}}_{0}$ corresponding to continuous SAS events.

Since the distribution of AHI in the dataset is non-normal, an AHI transformation unit is placed before the first BLS unit to perform a logarithmic transformation on the AHI labels of the training set, as shown in Equation (4):

(4) $$Y^{\prime }=log\left(Y+1\right)$$

where $Y^{\prime }$ is the transformed label and Y is the original AHI value. Since the AHI can be 0, 1 is added to the original AHI value, and $Y^{\prime }$ is mapped to the interval [0, *log*(*max*(*Y* + 1))], making the model easier to fit.

The output ${{\hat{AHI}}^{\prime }}_{S}$ of this MSBLS regression model is inverse-transformed to obtain ${\hat{AHI}}_{S}$, which, together with the AHI prediction value ${\hat{AHI}}_{g}$ of the first MSBLS in the AHI regression branch, are used for SAS prediction probability correction. Based on the difference between global information and local information, the subjects with a difference greater than 10 between ${\hat{AHI}}_{g}$ and ${\hat{AHI}}_{S}$ are selected as the samples to be corrected. Then, the difference in the number of respiratory events $Even_{d}$ is calculated as shown in Equation (5):

(5) $$Even_{d}=\left|{\hat{AHI}}_{g}-{\hat{AHI}}_{s}\right|*60$$

The values of the length of $Even_{d}$ starting from the top 10% of the subject’s probability sequence in descending order are transformed according to Equation (6):

(6) $${{\hat{P}}^{\prime }}_{0}\left(i\right)=\{\begin{array}{c} \left(1+v\right)*{{\hat{P}}^{\prime }}_{0}\left(i\right)\;\\ \left(1-v\right)*{{\hat{P}}^{\prime }}_{0}\left(i\right)\; \end{array}\begin{array}{c} {\hat{AHI}}_{g}-{\hat{AHI}}_{s}>0\\ {\hat{AHI}}_{g}-{\hat{AHI}}_{s}<0 \end{array}$$

where the transformation coefficient v is determined by the global–local difference between the subject and the position of the sample, as shown in Equation (7):

(7) $$v=0.5*Even_{d}*\frac{60}{T}*\frac{i}{N}$$

where T is the subject’s sleep duration in minutes, and i is the position of the sample.

The transformed ${{\hat{P}}^{\prime }}_{0}$ is used as the input of the SAS detection branch to replace the original ${\hat{P}}_{0}$ in the contextual BLS model.

#### 2.4.3. AHI Prediction Branch

In this paper, the AHI prediction branch and the SDFFU are used simultaneously to assist the SAS detection process. The AHI prediction branch uses global features for AHI estimation. This branch contains two MSBLS regression units, whose parameters and training strategies are the same as those of the MSBLS in the SDFFU. The output of the first MSBLS regression ${{\hat{AHI}}^{\prime }}_{g}$ is inverse-transformed and used to optimize the model in the SDFFU.

The AHI prediction value based on the final result of the SAS detection branch is employed as the incremental feature of the second MSBLS regression unit and is combined with the global features for AHI prediction. The output is inverse-transformed as shown in Equation (8) and then serves as the output of the AHI prediction branch of the model.

(8) $$Y=e^{\hat{Y\prime }}-1$$

## 3. Experiment Setup

In this paper, three different experiments were conducted. The first one is the baseline experiment, which involved performing 5-fold cross-validation on two datasets, respectively, to verify the overall performance of the model. The second one focuses on the different lengths of samples; we employed samples of different lengths to validate their performance on SAS detection and AHI prediction. The third one is an ablation experiment, which was used to verify the mutual promotion of the AHI prediction to the SAS detection in the model. All the above experiments used the same experimental conditions and parameters.

This paper mainly focuses on two tasks. The SAS detection was evaluated using five indicators: accuracy (ACC), Macro F1 (MF1), Cohen’s Kappa coefficient (Kappa), precision, and recall. For the AHI prediction task, the Mean Absolute Error (MAE) and Root Mean Square Error (RMSE) were used to measure the deviation between the AHI estimated value and the AHI label. The coefficient of determination $R^{2}$ and the Intra-class Correlation Coefficient (ICC) were used to evaluate the correlation between the AHI prediction sequence and the AHI label sequence. Let ${\hat{y}}_{i}$ represent the AHI prediction value and $\bar{y}$ represent the mean of the AHI label sequence. The calculation formulas for MAE and RMSE are as follows:

(9) $$\mathrm{MAE}=\frac{1}{m}\sum_{i=1}^{m}\left|y_{i}-{\hat{y}}_{i}\right|$$

(10) $$\mathrm{RMSE}=\sqrt{\frac{1}{m}\sum_{i=1}^{m}{\left(y_{i}-{\hat{y}}_{i}\right)}^{2}}$$

where $R^{2}$ is the ratio of the regression sum of squares to the sum of squares of deviations, as shown in Equation (10):

(11) $$R^{2}=\frac{\sum {\left({\hat{y}}_{i}-\bar{y}\right)}^{2}}{\sum {\left(y_{i}-\bar{y}\right)}^{2}}$$

Since only the model output was evaluated, the ICC (ICC (2, 1)) of the two-way random-effects model was adopted. This model is suitable for a single rater to measure the absolute consistency between two sequences, which can be expressed by Equation (12):

(12) $$\mathrm{ICC}=\frac{MS_{I}-MS_{E}}{MS_{I}+\left(O-1\right)MS_{E}+O*\left(\left(MS_{O}-MS_{E}\right)/n\right)}$$

where O is the number of sequences involved in the correlation evaluation, which is equal to 2 (the true AHI value and the predicted AHI), $MS_{I}$ is the mean square value of the AHI prediction sequence, $MS_{E}$ is the mean square error between the AHI prediction sequence and the AHI label sequence, and $MS_{O}$ is the mean square value of the AHI label sequence.

Meanwhile, to comprehensively evaluate the AHI prediction results, the SAS severity classification was performed based on the AHI prediction values. Taking 5, 15, and 30 as thresholds, the subjects’ SAS severity was divided into four categories according to the AHI label: no SAS, mild SAS, moderate SAS, and severe SAS. The SAS severity labels were constructed. In the same way, the SAS severity prediction results were obtained using the AHI prediction values. In addition, according to some practical requirements, the results of three groups of binary classifications were obtained using the SAS thresholds of 5, 15, and 30, respectively. ACC, MF1, Kappa, recall, and specificity were used to evaluate these SAS severity tasks.

All experiments in this paper were trained and tested on Matlab 2022b with an Intel(R) Core(TM) i7 6900K CPU configuration.

## 4. Results

### 4.1. The Overall Result of the SASBLS

The overall results of the five-fold cross-validation of the SASBLS on the two datasets are shown in Table 3. From the perspective of SAS event classification results, the ACC and MF1 of both datasets are higher than 82% and 72%, respectively, which proves that most SAS event samples were effectively detected. Generally speaking, due to the larger data size and the complex age structure of the subjects in the SHHS1 dataset, the results are relatively low. However, the overall performance of the model is relatively stable, indicating a certain degree of robustness.

Regarding the AHI regression results, the ICC of both datasets is 0.87, and the RMSE of the SHHS1 and SHHS2 datasets does not exceed 8, indicating a high consistency between the AHI prediction results of the model and the gold standard, with relatively small deviations. From the perspective of the MF1 and Kappa of the SAS severity classification, the predicted results of the two datasets are relatively even distributed, and there is no situation where the AHI values are overly concentrated.

For the AHI binary classification results, the performance of the two datasets shows a unified trend. The binary classification performance with a threshold of 15 is relatively poor, while the binary classification performance with a threshold of 30 is the best. The average accuracy of the two datasets is 92.69%, and the average recall is 98.09%, indicating that the SASBLS can accurately distinguish severe SAS patients from other subjects. The classification performance with a threshold of 15 is relatively low because some ambiguous features led to a decrease in the overall accuracy to 86%. When classifying with a threshold of 5, the recall is relatively low, especially for SHHS1. Due to the wide AHI distribution range (0–102), in the process of generating AHI through the SAS detection results, the AHI values of the subjects are generally higher than the gold standard. Although this causes misclassification of some subjects without SAS, it ensures the diagnosis rate of moderate and severe SAS patients, which has important application value.

**Figure 2:**
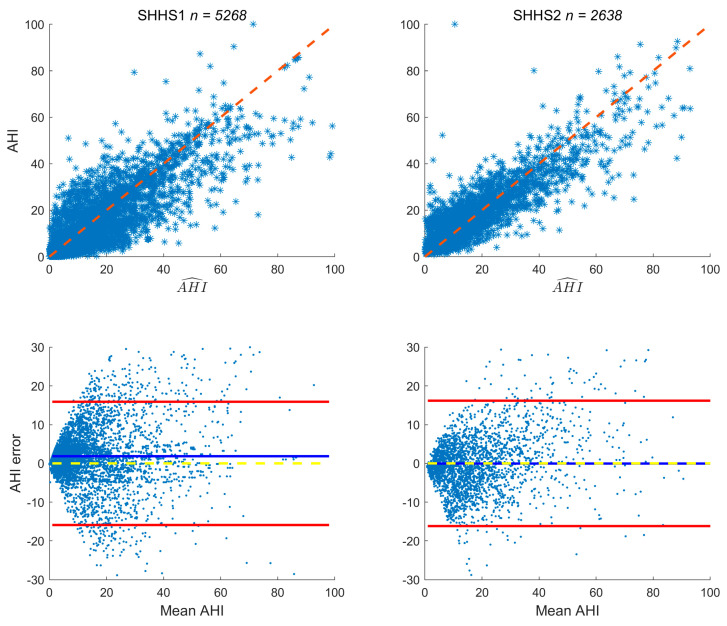
Correlation plots and Bland–Altman plots between the predicted AHI and the AHI labels for the two datasets. In the correlation plot, the red dashed line represents x=y. In the Bland–Altman plot, the blue solid line represents the mean of the differences between the predicted AHI and the AHI labels, the red error bars represent ±1.96 times the standard deviation of the differences between the predicted AHI and the AHI labels, and the yellow dashed line represents the zero-mean line. The correlation plots and the Bland–Altman plots corresponding to the two datasets are arranged in columns.

To further verify the AHI prediction results of the model, this paper analyzes the results using the correlation between the prediction results and the gold standard, as shown in Figure 2. Specifically, in the correlation plot, most samples of the two datasets are closely distributed on both sides of the diagonal line, proving a high consistency between the AHI prediction values and the gold standard. The existence of a few outliers does not significantly affect the overall consistency evaluation. In the lower part of the plot, the mean errors corresponding to the two datasets are both higher than the zero-mean line, clearly showing that the AHI prediction values are higher than the gold standard. This finding also confirms the binary classification results mentioned above. In comparison, there are more points deviating from both sides of the diagonal line in the results of SHHS1, which is also reflected in the number of outliers exceeding the upper and lower error lines. Although the deviation is relatively large compared with other datasets, considering its large sample size, this result is within an acceptable range.

### 4.2. Validation of the SAS Detection Performance of the Samples of Different Lengths

**Figure 3:**
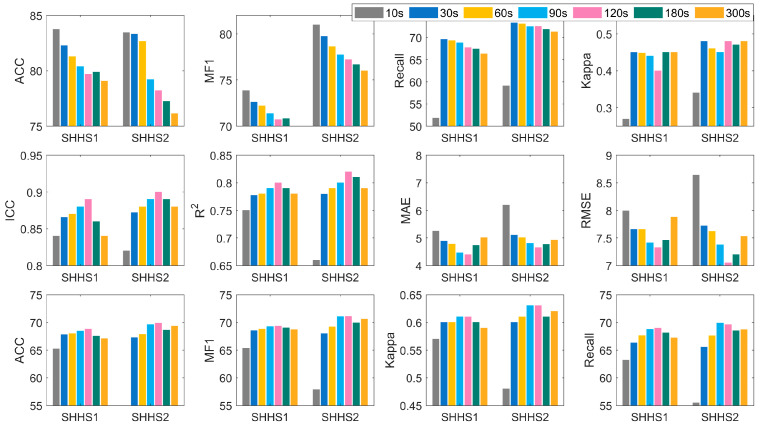
The AHI performance of samples with different lengths.

There is no unified standard for the sample lengths in the existing research on automatic classification of SAS events [21,27,28]. To investigate the influence of sample lengths on SAS event classification and AHI prediction, we carried out experiments using samples of varying lengths. In the experiment, time windows of 10 s, 60 s, 90 s, 120 s, 180 s, and 300 s are used for sample segmentation. The comparison results of AHI prediction and SAS severity classification are presented in Figure 3.

It should be noted that the composition of samples in the data space changes significantly with different lengths. Specifically, in the shorter samples, the sample imbalance is exacerbated. For example, the ratios of SAS-positive samples to normal samples for the 10 s, 30 s, and 60 s time slices are 24.4%, 33.82%, and 41.17%, respectively. Therefore, it is insufficient to evaluate the advantages and disadvantages of samples with different lengths solely based on the results of SAS detection, and it needs to be made by combining the SAS detection results and AHI prediction results.

From the perspective of SAS detection results, if only ACC and MF1 are considered, the results of 10 s samples are undoubtedly the best. However, when the recall is taken into account in the analysis, it is found that the accuracy of positive samples in 10 s samples is the lowest, being on average 18% lower than that of the baseline model. This is because of the relatively low proportion of positive samples in the data space of 10 s samples. And the 30 s samples have the best performance in SAS detection due to their moderate length.

From the perspective of AHI regression and SAS severity classification, as the sample length increases, the results of the corresponding model generally follow a parabolic trend. They start to increase from the 30 s time window, reach an extreme value at the 120 s time window, and then decline. For the results of the 120 s samples of the two datasets, the ACC, MF1, and Kappa of SAS severity increased by 1.35%, 1.69%, and 0.02, respectively, compared with the baseline model. The reason for this result is that a longer time window reduces the sample size, thereby reducing the errors caused by SAS detection. It is worth noting that due to the extremely low accuracy of positive samples in 10 s samples, their results in AHI regression and SAS severity classification are far inferior to those of samples with other lengths. The average result of the 300 s time window is lower than that of the baseline model as well. This is because an excessive sample length alters the sample composition; multiple SAS events that are relatively close to each other will be grouped into one sample.

Therefore, it is more reasonable to choose 30 s or 60 s samples. When the AHI prediction results are similar, the 30 s samples have better SAS detection effects and are more practical.

### 4.3. Ablation Experiment

**Figure 4:**
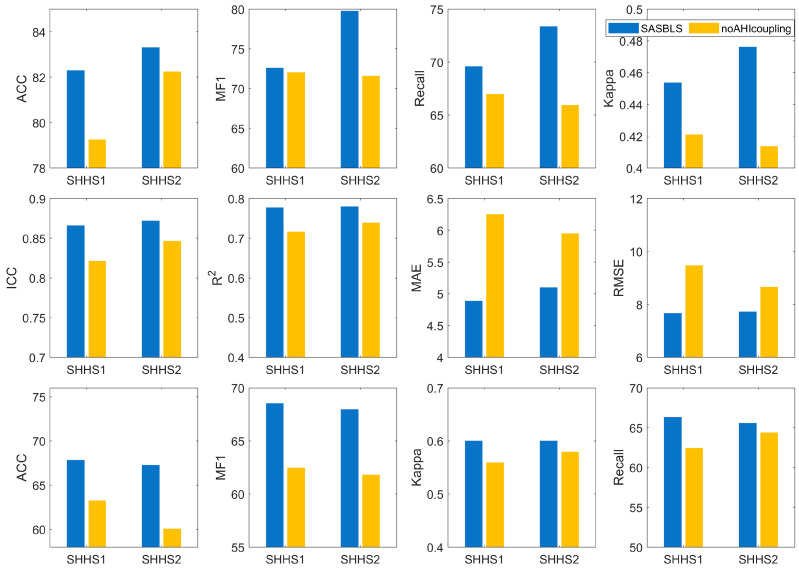
The results of the ablation experiment.

During the training process of the SASBLS, the SDFFU that fuses the differences between global features and sample features was used to optimize the model. We conducted an ablation experiment to explore the role of this method in the model. The model in the ablation experiment does not use global features. Instead, it only uses sample features for SAS event detection and AHI prediction. The comparison between the results of this model and those of the SASBLS is shown in Figure 4.

As can be seen from Figure 4, after fusing the differences between global features and sample features, the SASBLS significantly outperforms the ablation model in both SAS detection and AHI regression tasks. The average increments of ACC, MF1, recall, and Kappa in SAS detection are 2.06%, 6.38%, 5.76%, and 0.05, respectively, which significantly improve the overall results of SAS events. The ICC increases by an average of 0.04, and the RMSE decreases by an average of 1.37. In the SAS severity classification task, the average improvements in ACC, MF1, recall, and Kappa are 5.90%, 6.12%, 2.53%, and 0.03, respectively.

The results of the ablation experiment prove the effectiveness of the SDFFU in the model. Through the fusion of sample features and global features, the two tasks of SAS detection and AHI prediction promote each other. When compared with the ablation model, although the improvement in the ICC of the SASBLS is limited, it effectively reduces the RMSE of the predicted AHI, making the distribution of the prediction results more reasonable and closer to the real labels. This is reflected in the significant improvement in the accuracy of SAS severity classification.

## 5. Discussion

### 5.1. The Difference Between the Calculation AHI and the Regression AHI

The regression method was adopted in AHI prediction. The model results include the results of SAS event detection, and in the gold standard, the AHI value can be calculated by statistically analyzing the results of SAS detection. To compare the effectiveness of the two methods in the AHI regression task, this section compares the gold standard calculation method based on the results of SAS detection with the AHI regression results in the SASBLS. The results are shown in Table 4.

It is clear that compared with AHI regression, the AHI calculation based on the results of SAS event detection can hardly meet the application requirements. Its average ICC is only 0.71, and the average ACC and MF1 of SAS severity classification are 5.9% and 6.13% lower than those of the AHI regression model, respectively. This indicates that there are more outliers in the calculation method. This is because the calculation of AHI based on the subject’s data amplifies the errors in SAS event detection. In the SASBLS, the global features are constrained on a subject-by-subject basis through the SDFFU, thus reducing the error accumulation. Its function is similar to that of regularization in a classifier.

### 5.2. The Analysis of the Sample Size of 10 s and 30 s

According to the AASM manual, since the shortest length of an SAS event is 10 s, some studies on automatic SAS detection used a 10 s sample. However, such a sample is not suitable for SpO2-based SAS detection. Besides the sample composition analyzed above, there are two other reasons. First, regarding the stability of sample composition, taking SHHS1 as an example, events with a length of less than 15 s account for 30.67% of all events. If more than half of the window length is used as the standard for forming labels, then events shorter than 15 s will cause some samples to have oxygen desaturation characteristics but be labeled as negative. These samples pose great difficulties for model discrimination. Second, considering the characteristics of the SpO2 signal, the changes in SpO2 occur after the occurrence of an SAS event, and it is highly possible that there is no observable change in 10 s, leading to misjudgment. Therefore, when using the SpO2 signal for SAS detection, it is more appropriate to use samples of longer duration instead of 10 s. Taking the SHHS1 dataset as an example, the average length of SAS events is 21.6 s. Thus, choosing 30 s as the sample length is more reasonable. Furthermore, 30 s samples can be related to sleep staging, enabling a better observation of the relationship between SAS events and sleep stages.

### 5.3. Comparison with Other Studies

To further demonstrate the SAS detection performance of the model, in this section, the results of the SASBLS are compared with published SAS detection algorithms. Due to different experimental setups, the results in this section are obtained by reproducing the publicly available algorithms under the same experimental conditions as the SASBLS. The comparison of the results is shown in Table 5, where Thor and Abdo represent the chest–abdominal belt signals (Thor corresponds to the Thoracic channel, and Abdo corresponds to the Abdomen channel), AF represents the respiratory airflow channel (Air Flow), and ECG represents the electrocardiogram.

As can be seen from Table 5, the results of the SASBLS not only outperform models that also use a single-channel SpO2 but also exceed those of models using more channels. Among them, the most prominent are the MF1 and the recall. The advantage of recall clearly indicates that the SASBLS can effectively identify more SAS-positive samples, thus significantly increasing the SAS detection accuracy. Therefore, it can be said that the SASBLS has reached a state-of-the-art level in SAS detection.

In addition, in the results of published research, models using a single-channel signal are consistently inferior to those using multi-channel signals. After using respiratory airflow and chest–abdominal belt signals as inputs, the model performance has been significantly improved. This shows that chest–abdominal belt and airflow signals can effectively assist the model in discriminating positive SAS events. However, the performance of models using additional ECG has decreased. Considering that the original methods did not take into account the time effect of SAS events on different organs, it is speculated that the performance decline is caused by the phase difference between effective ECG features and respiratory signal features. Meanwhile, in order to showcase the most cutting-edge research achievements in OSA detection, some of the latest studies are listed in Table 5. These studies are in different experimental conditions from the SASBLS.

### 5.4. The Clinical Application Prospects of the Proposed Model

Given the ease of collecting SpO2 signals, OSA detection based on SpO2 holds considerable application prospects in clinical practice. The numbers of false-positive and false-negative samples in classification are directly related to the clinical application of the model. A high proportion of false-positive samples can lead to an overestimation of the severity of the subjects’ conditions, thus wasting medical resources. On the other hand, an excessive number of false-negative samples can seriously result in missed diagnoses of patients’ conditions, which will affect patients’ lives and health. Among them, the consequences caused by a relatively high number of false-negative samples are even more severe. Therefore, it is more important to minimize false-positive than false-negative samples.

In this paper, the overnight SpO2 information was used to optimize the SAS detection process, effectively reducing the false-positive and false-negative samples in the results. The diagnostic accuracy rate for patients with severe SAS reached 92.64%. However, it is worth noting that the overall predicted values of the AHI by the model are on the high side. This is a result of the model optimization being aimed at reducing false-negative samples, which leads to the situation that some healthy subjects are misjudged as having mild SAS. But this situation does not occur in the diagnosis of moderate and severe SAS cases. Therefore, we will further integrate clinical situations to optimize the model and provide a more reasonable and comprehensive solution for clinical applications.

## 6. Conclusions

This paper presents an SAS event detection model based on a single-channel SpO2 signal and the MSBLS. The model consists of a dual-branch architecture and takes 30 s samples as inputs, enabling it to concurrently execute the tasks of SAS event detection and AHI prediction. A feature fusion module was employed to enhance these two tasks. This module effectively fuses the differences between global and sample features to optimize the training process. The model achieved a state-of-the-art performance in the SAS prediction task. Additionally, the results demonstrate the influence of different sample lengths. The conclusion indicates that while samples with longer time durations can enhance the model’s AHI prediction performance when confronted with a more precise SAS detection task, 30 s samples prove to be more accurate.

## Figures and Tables

**Table 1:** The demographic of the used data.

Datasets	Subjects	Epochs	Age	Male Ratio	BMI	AHI	Avg. Length of SAS Events (s)
SHHS1	5268	5,257,566	62.54 ± 11.45	48%	27.92 ± 5.92	17.14 ± 18.47	21.6 ± 11.81
SHHS2	2638	2,979,845	62.42 ± 10.49	46%	28.32 ± 5.05	18.44 ± 16.37	22.83 ± 11.86

**Table 2:** The AHI prediction features.

Perspectives	Types of Features	Features
Signal features	Time domain	Mean, Median, MIN, STD, SpO2range, Px, Mx, ZC, ΔIx
	Frequency domain	PSD_total_, PSD_band_, PSD_ratio_, PSD_peak_
	Non-linear	ApEn, SampEn, PeEn, LZ complexity, DFA, CTMρ
Morphological features	-	PRSA_c_, PRSA_ad_, PRSA_s_, PRSA_sb_, PRSA_sa_
Desaturation features	Desaturation	ODIx, DL_μ_, DL_σ_, Dd_μ_, Dd_σ_, DS_μ_, DS_σ_, DA_μ_, DA_σ_, TD_μ_, TD_σ_
	Hypoxic burden	POD, AOD, CT_90_, CA_90_

**Table 3:** The overall results of the SASBLS.

SAS detection	**Datasets**	**ACC (%)**	**MF1 (%)**	**Kappa**	**Recall (%)**	**Precision (%)**
	SHHS1	82.28	72.59	0.45	69.58	70.68
	SHHS2	83.31	79.74	0.48	73.34	75.57
AHI		**ICC**	**R^2^**	**MAE**	**RMSE**	
	SHHS1	0.87	0.78	4.89	7.66	
	SHHS2	0.87	0.78	5.10	7.72	
SAS severity		**ACC (%)**	**MF1 (%)**	**Kappa**	**Recall (%)**	**Specificity (%)**
	SHHS1	67.82	68.54	0.60	66.31	88.19
	SHHS2	67.25	67.96	0.60	65.55	88.18
Health/SAS		**ACC (%)**	**MF1 (%)**	**Kappa**	**Recall (%)**	**Specificity (%)**
	SHHS1	87.51	76.83	0.59	55.53	94.21
	SHHS2	88.34	78.57	0.64	58.66	94.61
Health + mild/moderate and severe		**ACC (%)**	**MF1 (%)**	**Kappa**	**Recall (%)**	**Specificity (%)**
	SHHS1	86.22	86.10	0.76	92.70	78.08
	SHHS2	86.30	86.18	0.75	90.32	81.46
Other/severe		**ACC (%)**	**MF1 (%)**	**Kappa**	**Recall (%)**	**Specificity (%)**
	SHHS1	93.51	87.85	0.90	98.21	70.59
	SHHS2	91.77	85.46	0.88	97.96	64.56

**Table 4:** The comparison of 2 different methods.

Tasks	AHI Regression						SAS Severity Classification					
Metrics	ICC		R^2^		RMSE		ACC		MF1		Recall	
Methods	RE	CLA	RE	CLA	RE	CLA	RE	CLA	RE	CLA	RE	CLA
SHHS1	0.87	0.67	0.78	0.5	7.66	16.61	67.82	63.22	68.54	62.45	66.31	62.43
SHHS2	0.87	0.75	0.78	0.44	7.72	12.31	67.25	60.05	67.96	61.8	65.55	64.37

**Table 5:** The comparison of SAS detection results of the SASBLS and other studies.

Methods	Dataset	Used Signals	ACC (%)	MF1 (%)	Kappa	Recall (%)	Precision (%)
SASBLS	SHHS1	SpO2	82.28 *	**72.59**	**0.4** **5**	**69.58**	**70.68**
Ref. [19]		SpO2 + Thor + Abdo + AF	80.73	58.17	0.39	66.12	51.19
Ref. [19]		SpO2 + Thor + Abdo + AF + ECG	76.53	53.58	0.35	65.02	45.26
Ref. [19]		SpO2	78.05	55.73	0.38	59.12	52.71
Ref. [31]		SpO2	80.2	57.84	0.40	60.12	55.71
SASBLS	SHHS2	SpO2	83.31	**79.74**	**0.48**	**73.34**	**75.57**
Ref. [19]		SpO2 + Thor + Abdo + AF	**83.82**	58.56	0.40	64.09	53.91
Ref. [19]		SpO2 + Thor+Abdo + AF + ECG	79.11	55.73	0.34	63.25	49.81
Ref. [19]		SpO2	80.13	56.88	0.36	60.31	53.82
Ref. [26]	Apnea-ECG + SVUH *	SpO2	96.00	96.00	-	97.00	96.00
Ref. [22]	MESA	PPG + SpO2	87.03	74.31	-	74.40	91.29

* The best performance results are in bold. SVUH stands for the St. Vincent’s University Hospital dataset.

## Data Availability

The data are publicly available at https://www.sleepdata.org/datasets/shhs.
